# Amelioration for an ignored pitfall in reference gene selection by considering the mean expression and standard deviation of target genes

**DOI:** 10.1038/s41598-022-15277-5

**Published:** 2022-07-01

**Authors:** Ghazal Esfandiarpour, Mohammad Mokhtari, Seyed-Morteza Javadirad, Mohsen Kolahdouzan, Ahmed Almuslimawi

**Affiliations:** 1 Department of Cell and Molecular Biology and Microbiology, Faculty of Biological Science and Technology, University of Isfahan, Isfahan, Iran; 2 Department of Molecular Genetics, Faculty of Biological Sciences, Tarbiat Modares University, Tehran, 14115-111 Iran; 3 Department of Surgery, School of Medicine, Isfahan University of Medical Sciences, Isfahan, Iran

**Keywords:** Computational biology and bioinformatics, Molecular biology, Molecular medicine

## Abstract

Routine tissue-specific reference genes are often used in expression studies, but target genes are not taken into account. Using the relative RT-qPCR approach, we evaluated the expression of three target genes. At the same time, meta-analyses were conducted in various ethnic groups, genders, and thyroid cancer subtypes. When eight common reference genes were examined, it was discovered that some of them not only lacked consistent expression but also had considerable expression variance. It is worth noting that while choosing a reference gene, the mean gene expression and its standard deviation should be carefully addressed. An equation was developed based on this, and it was used to perform statistical analysis on over 25,000 genes. According to the subtype of thyroid cancer and, of course, the target genes in this investigation, appropriate reference genes were proposed. The intuitive choice of *GAPDH* as a common reference gene caused a major shift in the quantitative expression data of target genes, inverting the relative expression values. As a result, choosing the appropriate reference gene(s) for quantification of transcription data, and especially for relative studies of the expression of target gene(s), is critical and should be carefully considered during the study design.

## Introduction

Reference genes have been routinely used in gene expression analyses in traditional cancer studies^1,2^. Although one advantage of using reference genes is that their expression does not change under different physiological and experimental conditions^3,4^, numerous announcements have prohibited the use of routinely used reference genes blindly^5,6^. Furthermore, a groundbreaking analysis of RNA-seq data criticized the indiscriminate use of common reference genes^7^.

Glyceraldehyde-3-phosphate dehydrogenase (*GAPDH*) is a common reference genes in relative RT-qPCR experiments^8^. *GAPDH* was initially introduced as a suitable reference gene mainly due to its role in glycolysis; however, it is also involved in a variety of nuclear events such as transcription, RNA transport, DNA replication, apoptosis, nuclear translocation of proteins, and DNA repair^9–13^. The functional roles of *GAPDH* are not limited to cytoplasmic glycolysis, and more roles in the mitochondria and cytoskeleton have recently been discovered^14^. As a result, further investigation of *GAPDH* is required to determine its suitability for relative RT-qPCR data normalization. In this regard, we previously reported that *SYMPK* is a promising substituent reference gene among eight common reference genes, which include *B2M*, *TBP*, *ACTB*, *HPRT1, PYCR1, GUSB* and *GAPDH*^15^. To summarize, *SYMPK* had the lowest CqCV%, it was suggested by BestKeeper software in both normal and PTC tissues (r = 0.958 and 0.969, respectively) and *SYMPK*/*ACTB* had the lowest stability value = 0.209 according to the NormFinder algorithm. Finally, in addition to its statistical advantages, the *SYMPK* gene was proposed to normalize RT-qPCR data due to the lack of pseudogenes.

The target gene specificity and the sex-dependent behavior of reference genes were factors not previously considered in cancer studies. Fortunately, massive amounts of gene expression data are publicly available, allowing the selection of appropriate reference genes for any cancer study. As a result, we expanded on our laboratory findings in this study by conducting a precise and comprehensive bioinformatics meta-analysis. In our study population, routinely used reference genes were assessed in thyroid neoplasm subtypes in two scenarios: one that included patient sex consideration and the other that did not. *GAPDH* was not an appropriate reference gene in papillary thyroid cancer (PTC) tissues, as evidenced by our bioinformatics and lab-based experiments, because its expression was dependent on tumor subtypes.

We propose a novel approach for future cancer research: each target gene must have a unique reference gene(s). Then, using the NCBI gene expression omnibus, we created two gene lists: one for TCGA-PTC (with over 25,000 genes) and one for all thyroid neoplasm subtypes (GEO, with more than 6000 genes). An equation that emphasizes the mean and standard deviations of expression values from target genes was developed to accurately select reference genes.

## Results

**Figure 1:**
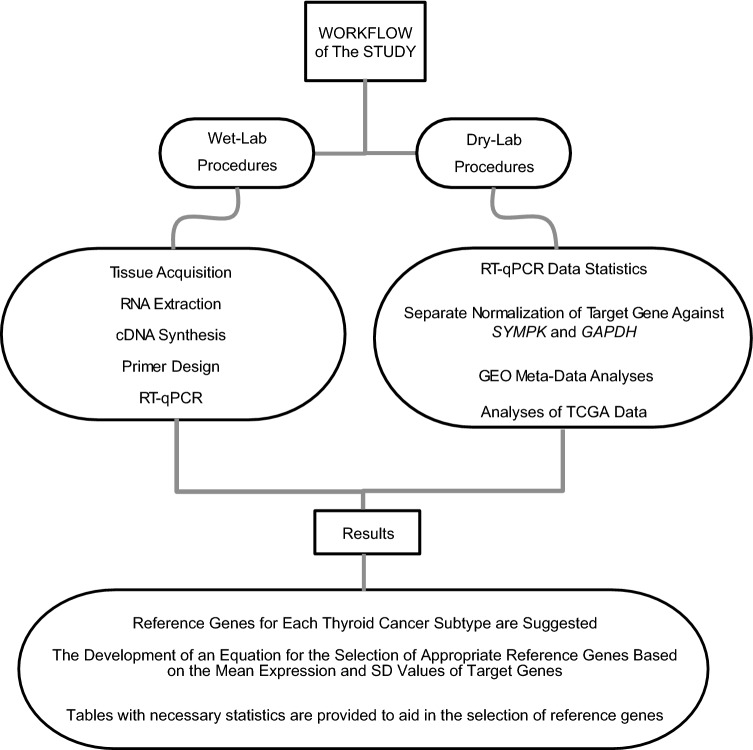
Workflow for performing bioinformatics analyses and laboratorybased investigations.

The workflow in Fig. 1 summarizes wet and dry lab procedures, including all laboratory experiments and in-silico analyses on datasets.

### Wet (laboratory) research

#### Quality and quantity of RNA

The mean absorbance ratios of wavelengths 260/280 and 260/230 were 1.96 ± 0.11 and 1.97 ± 0.06 for PTC tissues and their normal tissues, respectively. The intensity of 28S-rRNA bands was 1.5–2-times that of 18S-rRNA, indicating that the integrity of all extracted RNAs was satisfactory.

#### Target genes expression patterns

**Figure 2:**
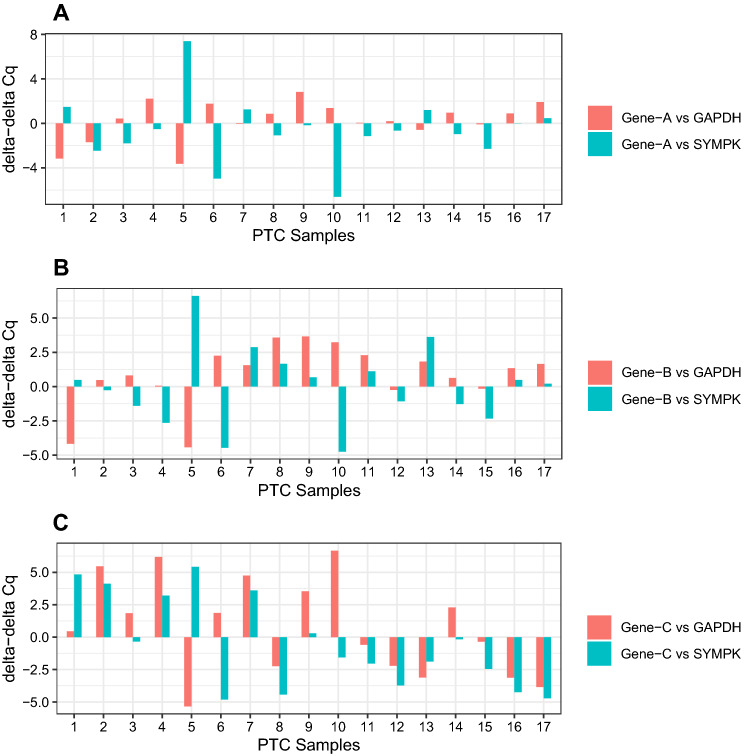
PTC tissues were compared to their adjacent normal tissues. Each target gene (gene A, gene B and gene C) was normalized once against "*GAPDH*" (red bands) and also a second time against "*SYMPK*" (blue bands). For Gene-A, 4 out of 17 samples (samples 2, 7, 16, and 17) show the same pattern after normalization against two different reference genes, while 13 samples (e.g. samples 1, 5, 6 and 10) show contradiction. For gene-B, 9 out of 17 samples and for gene-C, 5 out of 17 samples show contradiction. Positive and negative delta-delta Cq ratios respectively represent a target gene in PTC tissues that is down-regulated or over-expressed. Y-axis present deltadelta Cq ratios and X-axis show PTC samples.

Three target genes, NKX2-1 (Gene ID: 7080), RTRAF (Gene ID: 51637), and ETS1 (Gene ID: 2113), had their expression levels compared between PTC and adjacent normal tissues. To generalize the findings, these three target genes are now referred to as A, B, and C. The gene names were removed because they were unimportant to us, but their perplexing expression pattern after normalization with reference genes was. The expression of the target genes was normalized separately with the commonly used reference gene, *GAPDH*, as well as our recently approved *SYMPK* (Fig. 2). When normalized against *GAPDH* or *SYMPK*, Gene A showed contradiction for 13 out of 17 PTC samples, whereas only 4 samples (PTC samples 2, 7, 16, and 17) did not show contradiction. In PTC sample 1, Gene A was normalized against *GAPDH* (red bar) and a negative delta-delta Cq ratio was observed. A positive delta-delta Cq ratio was also observed for gene A just when the gene was normalized against *SYMPK* (blue bar). The same holds true for gene A in PTC samples 3, 4, 5, 6, 8, 9, 10, 11, 12, 13, 14, and 15. Therefore, only when *GAPDH* was replaced with *SYMPK* did gene A show 76.5% inconsistency. Dissimilitude was also observed when gene B (Fig. 2B, 52.9% differences) and gene C (Fig. 2C, 29.4% differences) were normalized against *GAPDH* and *SYMPK*. Therefore, when a specific PTC tissue was compared to its adjacent normal tissue, target gene could be reported as overexpressed or downregulated at the same time.

#### SYMPK and GAPDH expression in normal and PTC tissues

**Figure 3:**
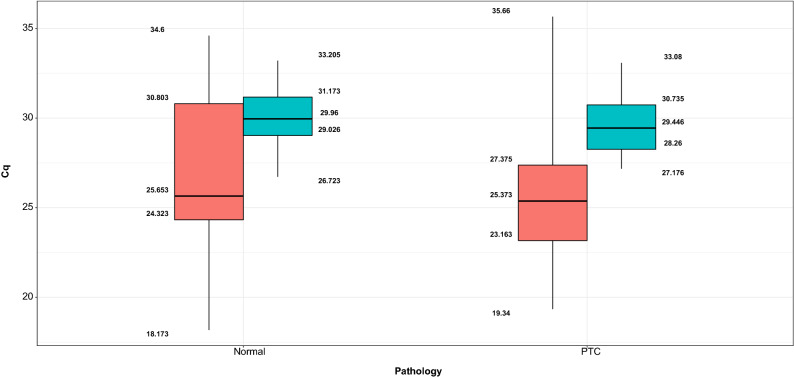
Expression of *SYMPK* and *GAPDH* in normal and PTC tissues. In normal tissues, *SYMPK* (blue bars) exhibited a smaller range of Cq values than *GAPDH* (red bars), and this was also true in PTC tissues, where *SYMPK* had less variance.

The best way to normalize RT-qPCR data is to pick a reference gene or genes that exhibit the least amount of variation in mRNA expression across all of the samples. *SYMPK* (Fig. 3, blue bars) showed a narrower range of Cq values than *GAPDH* (Fig. 3, red bars) in normal tissues, and the same was true in PTC tissues, where *SYMPK* had less variance.

#### Statistics on reference genes and target genes

Statistical analyses of target genes (A, B, and C) and reference genes (*GAPDH* and *SYMPK*) are presented in Table 1. *SYMPK* exhibited a lower SD = 1.74 and CqCV% = 5.84 in both the adjacent normal and the PTC tissues than *GAPDH* (SD = 4.26 and CqCV% = 16.32). To determine the differences in the expression values of *GAPDH* and *SYMPK* in the adjacent normal tissues as well as the PTC tissues, separate tissue statistics have been provided. In adjacent normal tissues, the mean Cq values of gene A (29.00) and gene B (28.74) were close to the mean Cq value of *SYMPK* (29.96) but far from the corresponding value of *GAPDH* (26.53). The same pattern was observed in PTC tissues, where the mean Cq value of *SYMPK* (29.59) was similar to that of genes A (28.44) and B (29.28), but not to *GAPDH* (25.69). In contrast to genes A and B, the mean expression of gene C in both adjacent normal tissues (26.24 and 26.53, respectively) and PTC tissue (25.8 and 25.69, respectively) was close to that of *GAPDH*. The *SYMPK* gene had a lower difference in expression between normal and PTC tissues (Cq = 29.96 and 29.59, respectively), whereas the *GAPDH* gene had a wider range of Cq values between normal and PTC tissues (Cq = 26.53 and 25.69, respectively). *GAPDH* gene expression, on the other hand, varied significantly more (3.85 < SD < 4.59) than target genes (2.65 < SD < 3.71). *SYMPK*, which had the lowest SD and CqCV% values in adjacent normal tissues, PTC tissues, and both tissues, was a better reference gene than *GAPDH*.

**Table 1:** Statistics for laboratory-collected RT-qPCR data.

Sample type	Gene name	Maximum	Minimum	SD	Mean	CV%
Normal + PTC	GAPDH	35.66	18.17	4.26	26.11	16.32
	SYMPK	33.20	26.72	1.74	29.77	5.84
Normal	GAPDH	34.60	18.17	4.59	26.53	17.32
	SYMPK	33.21	26.72	1.84	29.96	6.15
	Gene-A	35.18	21.29	3.37	29.00	11.64
	Gene-B	35.79	22.70	3.39	28.74	11.82
	Gene-C	31.74	16.96	3.71	26.24	14.14
PTC	GAPDH	35.66	19.34	3.85	25.69	15.00
	SYMPK	33.08	27.17	1.62	29.59	5.48
	Gene-A	34.53	23.22	2.65	28.44	9.35
	Gene-B	34.91	23.92	3.14	29.28	10.75
	Gene-C	31.08	21.08	2.76	25.81	10.70

For each target and reference genes, statistical parameters from laboratory experiments such as maximum, minimum, SD, mean and CV% are listed. *GAPDH* has the highest SD and CV% values in both PTC and adjacent normal tissues,while *SYMPK* has the lowest.

*SD* standard deviation, *CV* correlation of variation.

### Dry (bioinformatics) research

#### Inter-subtype comparisons

Fourteen microarray datasets with expression and phenotype data (Supplementary Table S2) were downloaded and cleaned (Materials and Methods). Because FVPTC (follicular variants of PTC) is the most common variant of PTC, FVPTC and PTC samples were analyzed as a single phenotypic group. For 6331 genes held in common, 520 samples were compiled, including 116 normal, 38 FTA (follicular thyroid adenoma), 246 PTC, 39 FTC (follicular thyroid carcinoma), 27 PDTC (poorly differentiated thyroid carcinoma), 52 ATC (anaplastic thyroid carcinoma), and 2 MTC (medullary thyroid carcinoma).Microarray probes were matched to corresponding genes, mean expression values for a probe set were calculated for each gene, and the data was subjected to “removeBatchEffect” (Supplementary Figs. S1 and S2).

**Figure 4:**
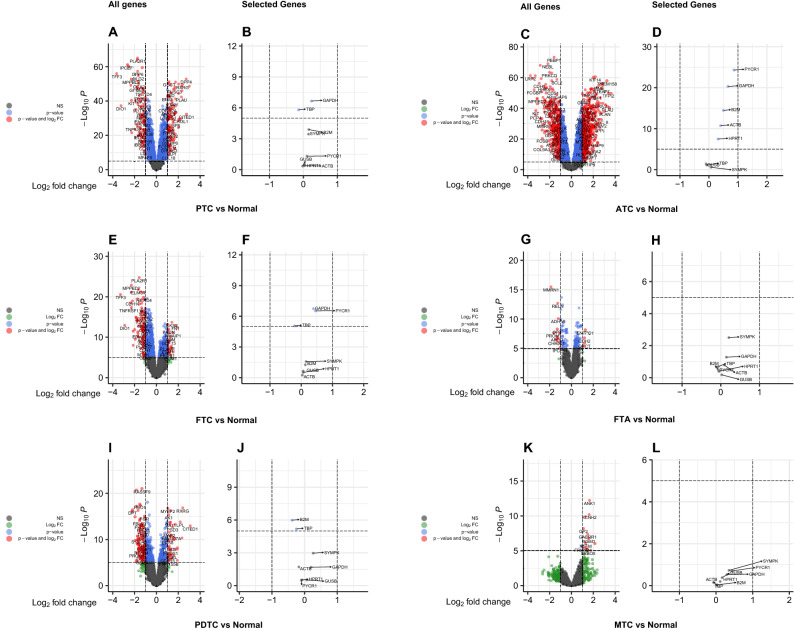
Volcano plots of differentially expressed genes and selected reference genes in each subtype in a microarray inter-subtype meta-analysis. (**A**) all genes and (**B**) selected reference genes of PTC versus normal analysis. (**C**) all genes and (**D**) selected reference genes of ATC versus normal analysis. (**E**) all genes and (**F**) selected reference genes of FTC versus normal analysis. (**G**) all genes and (**H**) selected reference genes of FTA versus normal analysis. (**I**) all genes and (**J**) selected reference genes of PDTC versus normal analysis. (**K**) all genes and (**L**) selected reference genes of MTC versus normal analysis.

The expression levels of eight common reference genes were compared in two ways: between normal tissues and each subtype of thyroid cancer, as well as between subtype (Table 2). *GAPDH* and *SYMPK* had effect sizes (ES) of 0.235 and 0.151 for the PTC subtype, respectively, when compared to normal tissues; however, the ES of *GAPDH* was statistically significant (p = 0.0020). *GAPDH* had statistically significant ES values in both the FTC (p = 0.0012) and ATC (p = 3.19E−17) subtypes. Furthermore, *GAPDH* had higher ES values than *SYMPK* in ATC (0.652 vs 0.070 respectively) and FTC (0.389 vs 0.154, respectively) subtypes. Other subtypes, such as FTA, PDTC, and MTC, showed negligible differences between *GAPDH* and *SYMPK* expression. *GUSB* (− 0.024), *ACTB* (0.032), and *HPRT1* (0.037) were the three most ideal reference genes in the PTC subtype, with the lowest insignificant ES (Fig. 4A,B). The best three reference genes for other subtypes were *SYMPK* (0.070), *TBP* (− 0.076) and *GUSB* (− 0.098) in ATC (Fig. 4C,D); *ACTB* (0.036), *HPRT1* (0.063), and *GUSB* (0.064) in FTC (Fig. 4E,F); *GUSB* (0.023), *HPRT1* (− 0.052), and *TBP* (− 0.062) in FTA (Fig. 4G,H); *GUSB* (0.062), *HPRT1* (− 0.075), and *PYCR1* (− 0.102) in PDTC (Fig. 4I,J); *ACTB* (0.015), *B2M* (0.027), and *TBP* (− 0.070) in MTC (Fig. 4K,L).

**Table 2:** Analyses of differential expression between normal tissues and thyroid cancer subtypes, as well as inter-subtype comparisions.

Tissues to be compared (number)	Gene name	ES	FWER
FTA (38) vs Normal (116)	GAPDH	0.144	1
	SYMPK	0.207	1
	GUSB	0.023	1
	ACTB	0.091	1
	TBP	− 0.062	1
	B2M	− 0.069	1
	HPRT1	− 0.052	1
	PYCR1	− 0.108	1
PTC (246) vs Normal (116)	GAPDH	0.235	0.0020
	SYMPK	0.151	1
	GUSB	− 0.024	1
	ACTB	0.032	1
	TBP	− 0.143	0.0099
	B2M	0.159	0.8142
	HPRT1	0.037	1
	PYCR1	0.105	1
FTC (39) vs Normal (116)	GAPDH	0.389	0.0012
	SYMPK	0.154	1
	GUSB	0.064	1
	ACTB	0.036	1
	TBP	− 0.217	0.0553
	B2M	0.130	1
	HPRT1	0.063	1
	PYCR1	0.455	0.0019
MTC (2) vs Normal (116)	GAPDH	0.297	1
	SYMPK	0.352	1
	GUSB	0.164	1
	ACTB	0.015	1
	TBP	− 0.070	1
	B2M	0.027	1
	HPRT1	0.114	1
	PYCR1	0.433	1
PDTC (27) vs Normal (116)	GAPDH	0.197	1
	SYMPK	0.263	1
	GUSB	0.062	1
	ACTB	− 0.175	1
	TBP	− 0.254	0.0415
	B2M	− 0.387	0.0065
	HPRT1	− 0.075	1
	PYCR1	− 0.102	1
ATC (52) vs Normal (116)	GAPDH	0.652	3.19E−17
	SYMPK	0.070	1
	GUSB	− 0.098	1
	ACTB	0.401	1.17E−07
	TBP	− 0.076	1
	B2M	0.497	2.23E−11
	HPRT1	0.314	**0.0002**
	PYCR1	0.401	1.17E−07
ATC (52) vs PTC (246)	GAPDH	0.416	1.11E−07
	SYMPK	− 0.089	1
	GUSB	− 0.032	1
	ACTB	0.369	8.22E−08
	TBP	− 0.001	1
	B2M	0.337	1.94E−05
	HPRT1	0.276	0.0006
	PYCR1	0.755	1.96E−19
ATC (52) vs FTC (39)	GAPDH	0.262	1
	SYMPK	− 0.083	1
	GUSB	− 0.162	1
	ACTB	0.365	0.0069
	TBP	0.140	1
	B2M	0.366	0.0189
	HPRT1	0.250	1
	PYCR1	0.405	0.3950
ATC (52) vs MTC (2)	GAPDH	0.354	1
	SYMPK	− 0.282	1
	GUSB	− 0.262	1
	ACTB	0.386	1
	TBP	− 0.005	1
	B2M	0.462	1
	HPRT1	0.199	1
	PYCR1	0.427	1
ATC (52) vs PDTC (27)	GAPDH	0.454	0.0115
	SYMPK	− 0.192	1
	GUSB	− 0.160	1
	ACTB	0.576	6.91E−08
	TBP	0.177	1
	B2M	0.883	1.75E−18
	HPRT1	0.388	0.0087
	PYCR1	0.962	8.85E−13
ATC (52) vs FTA (38)	GAPDH	0.507	2.49E−05
	SYMPK	− 0.137	1
	GUSB	− 0.121	1
	ACTB	0.309	0.2430
	TBP	− 0.014	1
	B2M	0.566	1.02E−08
	HPRT1	0.365	0.0020
	PYCR1	0.968	2.96E−16
PDTC (27) vs FTA (38)	GAPDH	0.052	1
	SYMPK	0.055	1
	GUSB	0.039	1
	ACTB	− 0.266	1
	TBP	− 0.191	1
	B2M	− 0.317	1
	HPRT1	− 0.022	1
	PYCR1	− 0.005	1

The levels of expression of eight common reference genes are compared between normal tissues and each thyroid cancer subtype. Differential expression was calculated using a linear model: expression ~ tumor, where tumor was a binary variable (tumor vs. normal). The *GAPDH* gene is expressed differentially in PTC, FTC and ATC tissues than in normal tissues. Meaningful ES values for *GAPDH*, *TBP* and *B2M* are recorded in PTC tissues. *GAPDH*, *PYCR1* and *TBP* have significant ES values in FTC tissues. The ES values of *B2M* and *TBP* are significant in PDTC tissues. In ATC tissues, *GAPDH*, *ACTB*, *B2M*, *HPRT1* and *PYCR1* all have a completely significant ES value. When compared to normal tissues, *SYMPK* gene retained its capability as a potential reference gene in all subtypes. The second section compares gene expression between undifferentiated (ATC subtype) and all other subtypes. A linear model was used to calculate differential expression: expression ~ tumor, where tumor was a binary variable (undifferentiated vs differentiated). *GAPDH*, with the except of FTC, shows significant differential expression between ATC and all other subtypes. Gene expression analyses are also carried out between poorly differentiated tissues (PDTC subtype) and all other differentiated subtypes (FTA, PTC, FTC, MTC). A linear model was used to calculate differential expression: expression ~ tumor, where tumor was a binary variable (poorly differentiated vs differentiated). There is no evidence of significant differential expression of any of the reference gene. For clarity, only PDTC versus FTA comparison is reported, and all other comparisons are omitted.

*ES* effect size, *FWER* family-wise error rate.

The inter-subtype analysis was divided into two parts: the first assessed the differential expression of reference genes between undifferentiated (ATC) subtype and all other subtypes, and the second part was devoted to assessing the differential expression of reference genes between the poorly differentiated (PDTC) subtype and differentiated subtypes (FTA, PTC, FTC, MTC). *GAPDH* had statistically significant differential expression between ATC and all other subtypes, with the exception of FTC (0.262) and MTC (0.354). When undifferentiated-ATC tissues were compared to differentiated-PTC tissues, the genes *GAPDH*, *ACTB*, *B2M*, *HPRT1*, and *PYCR1* were found to be significantly expressed. The same results were obtained when comparing undifferentiated-ATC tissues to poorly differentiated-PDTC tissues. A gene expression analysis was also performed to compare PDTC to other differentiated subtypes, and none of the reference genes were statistically significant. As a result, only a comparison of PDTC with FTA was reported in Table 2 and the others were omitted.

#### Intra-sex analyses, as well as sex-subtype interactions

Intra-sex analysis was performed to determine the differentially expressed reference genes in each of the two sexes, and the interaction of sex and subtype was investigated using factorial designs (Table 3). We dealt with 253 samples, including 44 normal, 15 FTA, 119 PTC, 24 FTC, 27 PDTC, and 24 ATC, after 6 out of 14 datasets failed to offer detailed information regarding the sex of the patients. We did not have any FTA-male samples, and no MTC subtype samples were left. Most of the reference genes did not reveal statistically significant differences in expression in intra-sex analysis. The only exceptions were ATC-women, who had statistically different expression of *B2M* (ES = 0.536, p = 0.0175) and *PYCR1* (ES = 0.900, p = 0.0290) genes. The ES value of *GAPDH* was higher in females than males in PTC subtype (ES = 0.222 vs ES = 0.028 respectively), but the difference was not statistically significant (ES.Female–ES.Male = 0.194, p = 1), according to the interaction analysis. There were also differences in the expression of some other reference genes between females and males (e.g. *TBP* in ATC and *B2M or GUSB* in FTC), but using a factorial design to calculate the differences in differential expression revealed no significant differences in the expression of these two genes (p = 1 and 1 or 0.4560, respectively).

**Table 3:** Intra-sex analyses, as well as sex-subtype interaction.

Sample status (number)	Gene name	Female		Sample status (number)	Male		Interaction analysis of sex and subtype	
		ES	FWER		ES	FWER	ES.Female -ES.Male	FWER
PTC (76) vs. Normal (30)	GAPDH	0.222	1	PTC (43) vs. Normal (14)	0.028	1	0.194	1
	SYMPK	0.193	1		0.280	1	− 0.087	1
	GUSB	0.058	1		0.016	1	0.042	1
	ACTB	− 0.029	1		0.039	1	− 0.068	1
	TBP	− 0.108	1		− 0.084	1	− 0.024	1
	B2M	0.036	1		0.022	1	0.014	1
	HPRT1	0.077	1		− 0.006	1	0.083	1
	PYCR1	0.144	1		0.155	1	− 0.011	1
FTC (15) vs. Normal (30)	GAPDH	0.086	1	FTC (9) vs. Normal (14)	0.431	1	− 0.345	1
	SYMPK	0.163	1		0.209	1	− 0.046	1
	GUSB	− 0.208	1		0.378	1	− 0.586	0.4560
	ACTB	− 0.150	1		0.149	1	− 0.299	1
	TBP	− 0.133	1		− 0.286	1	0.153	1
	B2M	− 0.010	1		0.316	1	− 0.326	1
	HPRT1	0.114	1		0.113	1	0.001	1
	PYCR1	0.351	1		0.563	1	− 0.212	1
PDTC (19) vs. Normal (30)	GAPDH	0.209	1	PDTC (8) vs. Normal (14)	− 0.114	1	0.323	1
	SYMPK	0.205	1		0.589	1	− 0.384	1
	GUSB	0.208	1		− 0.151	1	0.359	1
	ACTB	− 0.243	1		− 0.219	1	− 0.024	1
	TBP	− 0.347	1		0.084	1	− 0.431	1
	B2M	− 0.542	1		− 0.328	1	− 0.214	1
	HPRT1	− 0.072	1		− 0.074	1	0.002	1
	PYCR1	− 0.065	1		− 0.245	1	0.180	1
ATC (16) vs. Normal (30)	GAPDH	0.319	1	ATC (8) vs. Normal (14)	0.308	1	0.011	1
	SYMPK	0.166	1		0.094	1	0.072	1
	GUSB	0.013	1		− 0.212	1	0.225	1
	ACTB	0.209	1		0.447	1	− 0.238	1
	TBP	0.004	1		0.198	1	− 0.194	1
	B2M	0.536	0.0175		0.686	0.1078	− 0.150	1
	HPRT1	0.181	1		0.395	1	− 0.214	1
	PYCR1	0.900	0.0290		0.480	1	0.420	1
FTA (15) vs. Normal (30)	GAPDH	0.222	1	FTA (0) vs. Normal (14)	NA	NA	NA	NA
	SYMPK	0.193	1		NA	NA	NA	NA
	GUSB	0.058	1		NA	NA	NA	NA
	ACTB	− 0.029	1		NA	NA	NA	NA
	TBP	− 0.108	1		NA	NA	NA	NA
	B2M	0.035	1		NA	NA	NA	NA
	HPRT1	0.077	1		NA	NA	NA	NA
	PYCR1	0.144	1		NA	NA	NA	NA

Using three scenarios, the expression levels of eight common reference genes are compared between normal tissues and each subtype of thyroid cancer. In the first scenario, a linear model was used to calculate differential expression in females: expression ~ tumor, where tumor was a binary variable (tumor vs. normal). In the second scenario, male differential expression was calculated using the same linear model as in the first. In the third scenario, the differential expression of differences was calculated using a complex linear model: expression ~ group, where group was a single factor made up of sex and subtypes. As a result, the binary variable was female vs. male, and the contrast was (female.tumor-female.normal)—(male.tumor-male.normal). For intra-sex and interaction analyses, ES and FWER are presented separately. The interaction could not be calculated because there was no male with FTA subtype.

*ES* effect size, *FWER* family-wise error rate.

**Figure 5:**
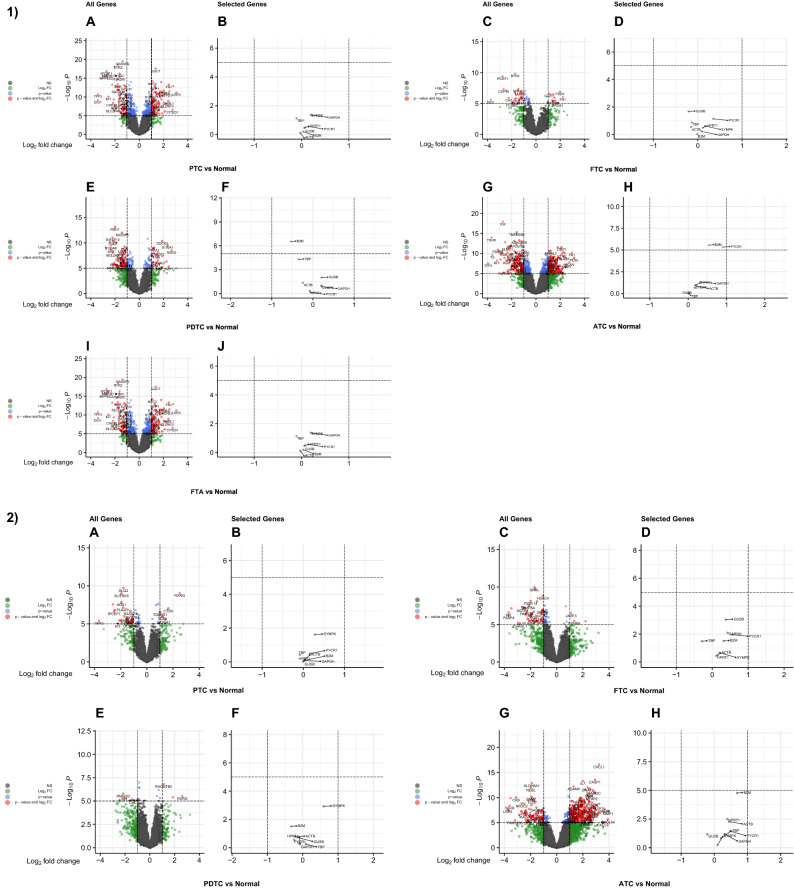
(1) Volcano plots of differentially expressed genes and selected reference genes in female samples in a microarray meta-analysis. (**A**) all genes and (**B**) selected reference genes of PTC versus normal analysis. (**C**) all genes and (**D**) selected reference genes of FTC versus normal analysis. (**E**) all genes and (**F**) selected reference genes of PDTC versus normal analysis. (**G**) all genes and (**H**) selected reference genes of ATC versus normal analysis. (**I**) all genes and (**J**) selected reference genes of FTA versus normal analysis. (2) Volcano plots of differentially expressed genes and selected reference genes in male samples in a microarray meta-analysis. (**A**) all genes and (**B**) selected reference genes of PTC versus normal analysis. (**C**) all genes and (**D**) selected reference genes of FTC versus normal analysis. (**E**) all genes and (**F**) selected reference genes of PDTC versus normal analysis. (**G**) all genes and (**H**) selected reference genes of ATC versus normal analysis.

The ES of reference genes were depicted in females and males based on their subtypes (Fig. 5-1,2 respectively). *ACTB* was the best reference gene in women with PTC (Fig. 5-1A\B) and FTA (Fig. 5-1I\J) subtypes, while *B2M* was the best in FTC-women (Fig. 5-1C\D), *PYCR1* was the best in PDTC-women (Fig. 5-1E\F), and *TBP* was the best in ATC-women (Fig. 5-1G\H). In males with PTC (Fig. 5-2A\B), PDTC (Fig. 5-2E\F), and FTC (Fig. 5-2C\D), *HPRT1* was the best reference gene, while *SYMPK* was the best in males with ATC (Fig. 5-2G\H).

#### Intra-subtype, inter-sex analysis

**Figure 6:**
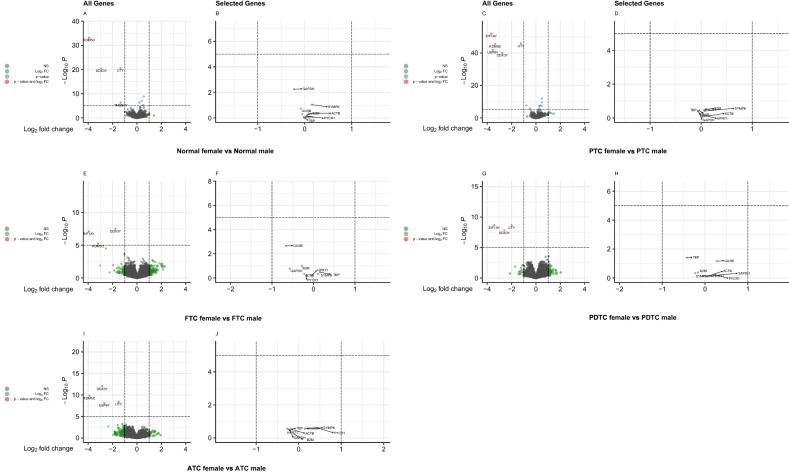
Volcano plots of differentially expressed genes and selected reference genes in a microarray meta-data based on intra-subtype and inter-sex analysis. (**A**) all genes and (**B**) selected reference genes of normal female versus normal male analysis. (**C**) all genes and (**D**) selected reference genes of PTC female versus PTC male analysis. (**E**) all genes and (**F**) selected reference genes of FTC female versus FTC male analysis. (**G**) all genes and (**H**) selected reference genes of PDTC female versus PDTC male analysis. (**I**) all genes and (**J**) selected reference genes of ATC female versus ATC male analysis.

With the exception of FTA, inter-sex analysis was performed within subtypes to determine the most appropriate reference gene in different pathological conditions (normal and subtypes, Table 4). *TBP*, *PYCR1*, and *B2M* were the best reference genes in normal tissues (Fig. 6A,B), while *ACTB*, *TBP*, and *HPRT1*, were the best ones in PTC subtypes (Fig. 6C,D). *HPRT1*, *SYMPK*, and *TBP* were the best genes for the FTC subtype (Fig. 6E,F), *HPRT1*, *ACTB* and *GAPDH* for the PDTC subtype (Fig. 6G,H), and *B2M*, *GUSB*, and *ACTB* for the ATC subtype (Fig. 6I,J).

**Table 4:** Combined analysis of intra-subtype and inter-sex microarray data.

Sample status (number)	Gene name	ES	FWER
Normal Female (30) vs. Male (14)	GAPDH	− 0.229	1
	SYMPK	0.152	1
	GUSB	− 0.081	1
	ACTB	0.079	1
	TBP	0.002	1
	B2M	0.046	1
	HPRT1	− 0.053	1
	PYCR1	0.043	1
PTC Female (76) vs. Male (43)	GAPDH	− 0.034	1
	SYMPK	0.065	1
	GUSB	− 0.039	1
	ACTB	0.011	1
	TBP	− 0.021	1
	B2M	0.060	1
	HPRT1	0.029	1
	PYCR1	0.032	1
FTC Female (15) vs. Male (9)	GAPDH	− 0.573	1
	SYMPK	0.106	1
	GUSB	− 0.667	1
	ACTB	− 0.220	1
	TBP	0.156	1
	B2M	− 0.280	1
	HPRT1	− 0.052	1
	PYCR1	− 0.169	1
PDTC Female (19) vs. Male (8)	GAPDH	0.095	1
	SYMPK	− 0.232	1
	GUSB	0.278	1
	ACTB	0.055	1
	TBP	− 0.428	1
	B2M	− 0.167	1
	HPRT1	− 0.052	1
	PYCR1	0.223	1
ATC Female (16) vs. Male (8)	GAPDH	− 0.217	1
	SYMPK	0.223	1
	GUSB	0.145	1
	ACTB	− 0.159	1
	TBP	− 0.192	1
	B2M	− 0.104	1
	HPRT1	− 0.267	1
	PYCR1	0.464	1

The results of microarray analyses that combine intra-subtype and inter-sex data are presented. Female tissues were compared to male tissues in normal tissues, and *TBP* had the lowest ES value (0.002). The best reference genes in PTC and ATC tissues, are *ACTB* and *B2M* with effect sizes of 0.011 and − 0.104, respectively. With an ES value of − 0.052, *HPRT1* was the best reference gene in both the FTC and the PDTC subtypes.

*ES* effect size, *FWER* family-wise error rate.

#### Microarray and RNA-seq data statistics

**Figure 7:**
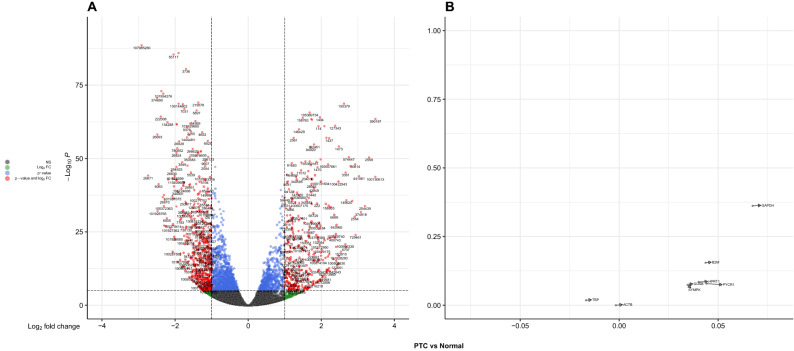
TCGA volcano plots of differentially expressed genes and selected reference genes in the PTC subtype. (**A**) all genes and (**B**) selected reference genes of PTC versus normal analysis.

The TCGA database was used to download raw expression counts of 560 samples, including 502 PTC and 58 normal tissues, and the statistics of this RNAseq data are shown in Table 5. *ACTB* (2.89), *GAPDH* (3.08), and *SYMPK* (3.25) were the top three genes in PTC tissues with the lowest CV% values. In normal tissues adjacent to PTC tissues, *SYMPK* (CV% = 2.84) was ranked after *GAPDH* (2.46) and *GUSB* (2.59). According to the differential expression of the reference genes (Table 6), the top three genes with the lowest ES values were *ACTB* (− 0.001), *TBP* (− 0.017), and *SYMPK* (0.034), respectively. *GAPDH* had the highest ES value = 0.06 among eight reference genes (Fig. 7).

**Table 5:** TCGA dataset statistics for eight selected reference genes.

Gene name	Normal maximum	Normal minimum	Normal SD	Normal mean	Normal CV%	PTC Maximum	PTC Minimum	PTC SD	PTC Mean	PTC CV%
GAPDH	16.72	14.72	0.39	15.94	2.46	18.21	14.40	0.50	16.48	3.08
SYMPK	13.52	11.50	0.35	12.58	2.84	14.02	10.88	0.41	12.70	3.25
GUSB	13.08	11.06	0.31	12.26	2.59	13.79	10.07	0.44	12.40	3.58
ACTB	19.22	16.70	0.64	17.84	3.63	18.99	15.71	0.50	17.57	2.89
TBP	10.54	8.29	0.45	9.94	4.54	10.86	6.62	0.47	9.68	4.94
B2M	18.95	15.21	0.79	17.13	4.64	19.73	12.63	1.07	17.41	6.16
HPRT1	11.43	9.95	0.30	10.64	2.85	12.06	8.79	0.47	10.79	4.43
PYCR1	9.47	5.67	0.77	8.17	9.45	12.39	2.80	1.36	8.31	16.48

TCGA database statistics are presented. *HPRT1* (0.30), *GUSB* (0.31) and *SYMPK* (0.35) genes had the lowest SD values in normal tissues, respectively. The genes with the lowest SD values in PTC tissues are *SYMPK* (0.41), *GUSB* (0.44), *TBP* and *HPRT1* (both 0.47).

*SD* standard deviation, *CV* correlation of variation.

**Table 6:** TCGA differential expression analysis in PTC samples.

Sample status	Gene name	Effect size	FWER
PTC vs. Normal	GAPDH	0.067	1
	SYMPK	0.035	1
	ACTB	− 0.002	1
	GUSB	0.036	1
	B2M	0.044	1
	TBP	− 0.017	1
	HPRT1	0.040	1
	PYCR1	0.044	1

PTC samples from the TCGA dataset were analysed for differential expression. *ACTB* (ES = − 0.002), *TBP* (ES = -0.017) and *SYMPK* (ES = 0.035) are the three most stable reference genes in PTC tissues.

*ES* effect size, *FWER* family-wise error rate.

Table 7 shows statistics for microarray pooled data from adjacent normal tissues and each thyroid cancer subtype. While *GAPDH* was ranked fifth (3.37), the genes with the lowest CV% values in normal tissues were *GUSB* (2.77), *B2M* (2.86), and *SYMPK* (3.10), respectively. *GUSB* (2.48), *GAPDH* (2.56), and *ACTB* (2.86) had the lowest CV% values in PTC tissues, followed by *SYMPK* (3.38).

**Table 7:** GEO microarray dataset statistics for eight selected reference genes.

Sample type	Gene Name	Maximum	Minimum	SD	Mean	CV%
Normal	GAPDH	12.13	9.49	0.38	11.25	3.37
	SYMPK	11.50	9.13	0.31	10.21	3.10
	GUSB	11.46	9.72	0.29	10.72	2.776
	ACTB	8.71	6.45	0.23	7.52	3.16
	TBP	6.57	4.30	0.36	5.84	6.22
	B2M	7.60	6.29	0.20	7.13	2.86
	HPRT1	7.29	5.02	0.29	6.44	4.57
	PYCR1	6.40	4.63	0.28	5.66	4.99
PTC	GAPDH	12.17	9.65	0.28	11.28	2.56
	SYMPK	11.62	9.16	0.35	10.37	3.38
	GUSB	11.66	9.68	0.27	10.95	2.48
	ACTB	8.16	6.79	0.21	7.50	2.86
	TBP	6.82	5.11	0.27	5.95	4.64
	B2M	7.71	6.41	0.20	6.99	2.91
	HPRT1	7.38	4.93	0.28	6.48	4.35
	PYCR1	8.08	4.42	0.35	5.81	6.14
FTA	GAPDH	11.96	10.27	0.27	11.34	2.42
	SYMPK	10.55	9.58	0.22	10.14	2.20
	GUSB	11.49	9.85	0.33	10.86	3.10
	ACTB	8.15	6.85	0.29	7.55	3.95
	TBP	6.83	4.65	0.52	5.73	9.11
	B2M	7.72	6.68	0.23	7.07	3.27
	HPRT1	6.98	5.90	0.26	6.39	4.13
	PYCR1	6.41	5.22	0.24	5.86	4.18
FTC	GAPDH	11.89	9.11	0.55	11.29	4.94
	SYMPK	11.55	8.94	0.45	10.34	4.36
	GUSB	12.48	7.86	0.80	11.10	7.27
	ACTB	8.34	6.45	0.47	7.59	6.22
	TBP	7.97	5.24	0.74	6.30	11.80
	B2M	7.55	6.14	0.31	6.91	4.55
	HPRT1	7.33	5.86	0.27	6.50	4.19
	PYCR1	6.81	5.24	0.29	5.81	5.12
MTC	GAPDH	11.30	11.23	0.049	11.27	0.44
	SYMPK	10.36	10.12	0.16	10.24	1.61
	GUSB	11.04	10.98	0.043	11.01	0.39
	ACTB	7.74	7.63	0.08	7.69	1.05
	TBP	6.46	6.08	0.26	6.27	4.28
	B2M	7.09	7.04	0.03	7.06	0.50
	HPRT1	6.71	6.39	0.22	6.55	3.44
	PYCR1	6.26	5.76	0.34	6.01	5.80
PDTC	GAPDH	11.73	9.58	0.45	11.08	4.08
	SYMPK	10.64	9.07	0.48	9.82	4.90
	GUSB	11.75	8.70	0.63	10.91	5.85
	ACTB	8.12	6.53	0.34	7.59	4.60
	TBP	7.83	3.89	0.93	5.74	16.35
	B2M	7.91	5.56	0.50	6.88	7.28
	HPRT1	7.24	5.55	0.34	6.37	5.35
	PYCR1	7.79	4.40	0.75	5.92	12.66
ATC	GAPDH	12.42	10.45	0.32	11.65	2.79
	SYMPK	11.92	9.62	0.46	10.71	4.37
	GUSB	12.56	9.27	0.50	11.37	4.41
	ACTB	8.61	6.52	0.41	7.43	5.52
	TBP	8.68	4.89	0.73	6.70	10.99
	B2M	8.49	6.37	0.37	7.05	5.37
	HPRT1	8.30	4.59	0.61	6.75	9.12
	PYCR1	7.43	5.01	0.45	5.73	7.98

Based on microarray datasets meta-analyses, statistics for eight reference genes were derived. *B2M* (0.20) in normal and PTC tissues, *SYMPK* (0.22) in FTA tissue, *PYCR1* (0.29) in FTC tissue, *B2M* (0.03) in MTC tissue, *ACTB* and *HPRT1* (0.34) in PDTC tissue, and *GAPDH* (0.32) in ATC tissue are the genes with the lowest SD values.

*SD* standard deviation, *CV* correlation of variation.

To facilitate use, the basic statistic for all 6331 genes in the GEO dataset (Supplementary Table S3) and all 25,705 genes in the TCGA dataset (Supplementary Table S4) were provided. These two tables compare the mean and standard deviation values of prospective target genes with the statistics of candidate reference genes.

## Discussion

In research and clinical detection, RT-qPCR is the gold-standard method for expression evaluation^16–18^. The advantageous of RT-qPCR include high sensitivity and specificity, speed of analysis, and real-time monitoring of results^8^. Nature protocols require that appropriate internal reference gene(s), formerly known as housekeeping genes, be validated prior to each study^19,20^. Historically, an ideal reference gene has minimally altered expression under various pathological and physiological conditions such as tumour type and patient sex. It must be free of pseudogene(s) and alternative splicing^15^. We previously investigated eight reference genes and discovered that *SYMPK* was more stably expressed than conventional reference genes (*GAPDH* and *ACTB*) and also lacked pseudogenes^15^. Ribosomal RNA (18S rRNA) is a highly recommended reference gene for RT-qPCR data normalization^21,22^. Unfortunately, 18S rRNA has at least three drawbacks: inhibition by mitomycin C^23^, absence in bulk high-throughput expression platforms, and a clear role in cancer development^24–28^ and prognosis^29^. We did not include 18S rRNA in our study due to the aforementioned facts and a previous report about its unstable expression^30^.

*GAPDH* and *SYMPK* were used as reference genes to normalize three candidate genes to better understand the consequences of using inappropriate reference genes. *GAPDH* was chosen because it is the most commonly used reference gene in molecular biology, and we previously reported it as the worst reference gene using NormFinder algorithm^15^. This is in line with a previous study that found *GAPDH* to be unsuitable for normalizing relative RT-qPCR data from bladder and colon cancer^31^. The gene did not meet the criteria of those authors (e.g. tissues stability, expression level above background, and lack of alternative splicing), so it was eventually ignored despite being ranked in colon cancer.

In this study, the expression of reference genes (*GAPDH* and *SYMPK*) was compared between normal and PTC tissues, *SYMPK* was found to be a better reference than *GAPDH* because it had less variability. Aside from the lack of alternative splicing, lower CqCV% values for *SYMPK* gene were obtained from relative RT-qPCR data in both normal and PTC tissues. The main point of contention is that *GAPDH* had a significantly higher SD than the target genes, a flaw that makes it decidedly inappropriate for mRNA expression normalization. We performed a meta-analysis on GEO microarray data combined with a comprehensive TCGA RNA-seq data analysis to increase the sample size, include all thyroid cancer subtypes, and involve both sexes. We discovered that *GAPDH* was significantly upregulated in PTC, FTC, and ATC, and as a result, the gene is unsuitable as a reference gene according to the microarray meta-analysis. *GAPDH* was found to be significantly upregulated at various stages of tumor differentiation. This idea suggests that *GAPDH* may be a key promoter of tumor aggressiveness, as previously reported by Chiche et al. in non-Hodgkin’s B lymphomas^32^. They proposed that the increased *GAPDH* levels activated the nuclear factor-κB gene, which in turn increased the activity of hypoxia-inducing factor-1α (*HIF-1α*). In this study, when FTA and ATC subtypes were compared, the expression of *HIF-1α* was also upregulated (ES = 0.497, p = 0.0001).

We provided separate tables to assist researchers in accurately selecting reference genes for their study designs. For example, if researchers want to study different subtypes, Table 2 provides a list of genes, and the gene with an ES closer to zero is the best fit for their research. Researchers could use Table 3 to include the gender of patients in an analysis, and the best genes are those with ES.Female -ES.Male closer to zero. Table 4 is the best reference when a specific subtype is required as well as the gender of the patients, with genes with ES values closer to zero serving as the best reference genes.

Furthermore, a discrepancy was discovered when each target gene was normalized against two different reference genes, *SYMPK* and *GAPDH*. (Fig. 2 and Table 8). We hypothesized that the differnce was occurred because of the overlap between the Cq values of target and reference genes. By overlapping, we mean that the Cq values of the reference and target genes are within the same range, and thus samples with positive ddCq mutually neutralize samples with negative ddCq, resulting in a change in the overall expression pattern of a target gene (Supplementary Figs. S3). To solve the issue arising, researchers should use Eq. (1) when they are trying to select reference genes.

1 $$ {\text{abs }}\left( {\mu {\text{T}} - \, \mu {\text{R}}} \right) \, \ge \, \left( {{3}\sigma {\text{T }}} \right) + \left( {{2}\sigma {\text{R}}} \right) $$

abs: absolute value, µ: mean, σ (SD): standard deviation, T: Target gene expression in each subtype, R: Reference gene expression in each subtype.

**Table 8:** Single PTC sample analysis using ddCq method.

Target gene vs. Reference gene	Numbers of PTC samples with negative ddCq	Numbers of PTC samples positive ddCq
Gene A vs. *SYMPK*	11	6
Gene A vs. *GAPDH*	6	11
Gene B vs. *SYMPK*	7	10
Gene B vs. *GAPDH*	4	13
Gene C vs. *SYMPK*	11	6
Gene C vs. *GAPDH*	8	9

Normalization of Gene A against SYMPK reveals that 11 of the 17 PTC samples have negative ddCq, while the remaining six have positive ddCq. When Gene A is normalized against GAPDH, the results are flipped, with six PTC samples exhibiting negative ddCq and eleven exhibiting positive ddCq. Gene B and Gene C also have opposing patterns. PTC samples with positive ddCq mutually neutralize PTC samples with negative ddCq, resulting in a change in the overall expression pattern of a target gene. Each PTC sample is compared with its adjacent normal tissue.ddCq: delta-delta Cq.

Consider the case where a reference gene has no variation in its expression (σ = 0) and a target gene has σ = 1. If the difference in mean expression between the target and the reference is at least three times the absolute value of the target gene's SD (3σT), the reference gene does not overlap with the target gene (Supplementary Fig. S4a). However, a reference gene with an SD value of 0.25 necessitates a difference of at least 3.5 units between the reference and target genes' mean expression values (Supplementary Fig. S4b). By doubling the SD value of the reference gene (from 0.25 to 0.5 and from 0.5 to 1), the mean expression values of the reference and the target genes must differ by 4 (Supplementary Fig. S4c) and 5 units (Supplementary Fig. S4d) respectively. To avoid overlap, we found that twice the absolute value of the SD of the reference gene (2σR) must also be considered for the calculation of the difference between the mean expression of the reference and the target genes. Therefore, it is possible to avoid overlaps between the expression values of reference gene and target gene and stop contradictory gene expression patterns by using Eq. (1).

For all expressed genes in GEO and TCGA, we provided tables with basic statistics, such as mean and SD (Supplementary Tables S3, S4). Our expression data could be a reliable estimate of any population for researchers to compare the mean and SD of desired genes in the above equation because our analyses include large sample sizes representing multiple ethnicities and subtypes in both sexes.

In conclusion, selecting reference gene(s) solely on the basis of specific tissues may result in inaccurate or misleading information. We questioned the common practice of selecting traditional reference genes. In a comprehensive investigation of thyroid cancer subtype, we discovered that *GAPDH* was significantly influenced by the aggressiveness of thyroid tumor subtypes. We created a new equation to help researchers choose the best reference gene(s) based on their desired target genes.

## Materials and methods

### Ethics statement

All patients who had PTC prior to surgery were given thorough explanations about sampling procedures, anonymous data publication, and rights of the subjects. All participants signed written informed consent forms. Tissues were not included in the study if any patient refuse to participate. This study was approved by the Isfahan University ethical committee's institutional review board (IR.UI.REC.1398.058). All experiments and procedures in this study, including but not limited to human participants, were carried out in accordance with the 1964 Helsinki Declaration and its subsequent amendments or comparable ethical standards.

### Human tissue acquisition

Seventeen PTC tissues and their adjacent normal tissues were taken from patients undergoing total or partial thyroidectomy et al. Zahra and Sina hospitals in Isfahan, Iran. Approximately 50 mg of freshly dissected PTC tissues and adjacent normal tissues were immediately submerged in 1 ml RNAlater, RNA Stabilization Reagent (Qiagen, Hilden, Germany) and incubated at 4 °C for 24 h per the manufacturer’s instructions. Tissue samples were then briefly centrifuged to remove any residual RNAlater before being stored at − 80 °C for further analysis. The hospital or third-party laboratories performed postoperative histopathological analyses and pathological approval. Pathological staging was reported using the American Joint Committee on Cancer Tumor-Node-Metastasis (TNM) staging system, 7th edition.

### RNA extraction and assessment

Total RNA was extracted from RNAlater-treated samples using a one-step RNA extraction reagent (Bio Basic, Markham, ON, Canada), as directed by the manufacturer. The concentration of isolated RNA was determined using a NanoDrop OneC spectrophotometer (Thermo Scientific, Waltham, MA, USA). A260/A280 and A260/A230 ratios were used to determine RNA purity. The integrity of the RNA was determined using 1.0% agarose gel electrophoresis.

### Complementary DNA (cDNA) synthesis

DNase I treatment (Thermo Scientific, Bremen, Germany) was used to remove residual genomic DNA contamination, as directed by the manufacturer. One microgram of total RNA was reverse transcribed in a total reaction volume of 20 μL using the Thermo Scientific RevertAid Reverse Transcriptase kit (Thermo Scientific, Bremen, Germany) according to the manufacturer’s instructions.

### Design of exon-junction primers

To avoid amplifying genomic DNA and/or heterogeneous nuclear RNA, all primers were exon junctioned. Beacon Designer 8.1 (Premier Biosoft International, Palo Alto, CA, USA) was used to design primers that span specific exons. Oligo 7 was used to recheck the primers for any unwanted secondary structure (Molecular Biology Insights, Colorado Springs, CO, USA). The NCBI-primer BLAST service was used to confirm the specificity of the designed primers. The melting temperature of the primers was validated using temperature gradient PCR (Sinaclon Bioscience, Tehran, Iran). All of the information on the primer pairs is presented in Supplementary Table S1.

### Relative RT-qPCR

In a Bio-Rad Chromo4 device (Bio-Rad, Hercules, CA, USA), a relative RT-qPCR reaction was performed using SYBR Green RealQ Plus 2 × Master Mix (Ampliqon, Odense, Denmark). The RT-qPCR reaction protocol consisted of (i) one cycle of enzyme activation and initial denaturation at 95 °C for 15 min, and (ii) 40 cycles of denaturation at 95 °C for 30 s, annealing for 30 s, and extension at 72 °C for 30 s. After each cycle, the plates were read. All relative RT-qPCR reactions were run in triplicate, with non-template control (NTC) per gene.

### Melt curve analysis

To assess the specificity of relative RT-qPCR, the melt curve was constructed by observing the gradual rise of temperature in 1 °C increments from 55 to 95 °C, followed by plate reading. The temperature (°C, x-axis) was plotted against the derivative of fluorescence change over temperature (y-axis).

### Gene expression analysis

Cq values were exported from the Bio-Rad Chromo4 thermocycler into Microsoft Excel (2013) for further analysis. The average of Cq values for reference and target genes in PTC tissues and adjacent normal tissues was calculated and the Livak method was used for normalization^2^. The delta Cq values were calculated by subtracting the Cq values of a reference gene and a target gene from each sample, and delta-delta Cq was determined by the difference between each PTC tissue and the average of delta Cq in adjacent normal tissues.

### Statistical analysis

Microsoft Excel 2013 (Microsoft, Redmond, WA, USA) was used to calculate qPCR fold change, maximum Cq, minimum Cq, standard deviation (SD), mean Cq, and correlation of variation (CqCV%, CqCV% = SD/mean × 100). CV% is a statistical measure that represents the relative dispersion of gene expression values in a dataset, regardless of the mean expression values of the genes. It is used to circumvent the problematic investigation of SD without considering the overall expression.

### Data collection

The GEO and The Cancer Genome Atlas (TCGA) databases were used to obtain microarray and RNAseq data, respectively. To scavenge any microarray expression data related to thyroid neoplasm, the GEO database was mined for the keywords “thyroid neoplasm”, “thyroid cancer”, and “thyroid carcinoma”. Exclusion criteria were used, and any data from species other than *Homo sapiens* was discarded. Cell lines, treatments, therapies, knocked-in and knocked-out models, and any dataset with incomplete phenotype information were excluded from further analysis. To reduce other biases, samples were collected from different countries and from people of various ethnicities. To compensate for the small sample size in different sexes and pathological subtypes, pooled data analyses were performed. As a result, 14 microarray datasets containing 520 samples were used in this study. FTA, PTC, FVPTC, FTC, MTC, PDTC, and ATC were among the thyroid neoplasms represented in the datasets. Microarray datasets are described in detail in supplementary Table S2.

### Pooled data analysis and calculation of effect size

Although the protocols for microarray and RNAseq analyses differed, the first step was to perform single dataset quality controls. Box plots were used to validate the log_2_ transformation and quantile normalization. Outlier detection was accomplished through the use of hierarchical clustering based on the Pearson correlation coefficient (PCC) as well as principal component analysis (PCA). The expression data from the outlier-removed datasets was compiled, the batch effect was removed with the Limma package's “removeBatchEffect” command, and a PCA plot was generated. The Limma package was used to analyze the pooled data, and the effect size (ES) was calculated. The family-wise error rate (FWER) “bonferroni” method was used to correct *P*-values. The effect size with FWER < 0.05 was deemed significant. The best reference genes had the lowest ES and a non-significant p-value. For inter-subtype analysis (subtypes-normal, undifferentiated-differentiated, poorly differentiated-differentiated), intra-sex analysis (subtypes-normal, separately in females and males), and intra-subtype/inter-sex analysis (females-males, separately in each subtype), two groups models were built. Interaction analysis was also performed between male and female, and a factorial design was used to estimate the impacts of the individuals' sex at various levels of the cancer subtypes ((female.tumor-female.normal)—(male.tumor-male.normal)).

The edgeR package was used to calculate logFC and FWER corrected *P*-values from TCGA raw read counts. *GAPDH* and *SYMPK* were two of the eight reference genes, with the remaining six being *GUSB*, *ACTB*, *B2M*, *TBP*, *PYCR1,* and *HPRT1*. For all the analyses, the software platform R 4.0.1 (R Foundation 3.6.2 for Statistical Computing, 2020, Austria) was used.

### GEO and TCGA datasets statistics

Using the RStudio environment, maximum, minimum, SD, mean, and CV% were calculated from the expression values of the selected genes in both the microarray pooled data and the TCGA. After compiling the expression data for each cancer subtype separately, statistical terms were calculated for each row representing each gene. A total of 6331 genes from microarray pooled data analysis output and 25705 genes from TCGA analysis output were statistically analyzed.

## Supplementary Information

Supplementary Information 1.

Supplementary Information 2.

Supplementary Information 3.

Supplementary Information 4.

Supplementary Information 5.

Supplementary Information 6.

Supplementary Information 7.

Supplementary Information 8.
