# Supplementary material for: Amelioration for an ignored pitfall in reference gene selection by considering the mean expression and standard deviation of target genes
Source: Sci Rep. 2022 Jul 1;12:11129. doi: 10.1038/s41598-022-15277-5 (PMC9249883; doi:10.1038/s41598-022-15277-5)
Supplement: Supplementary file 1 — Supplementary Information 1. [file 41598_2022_15277_MOESM1_ESM.pdf]

A

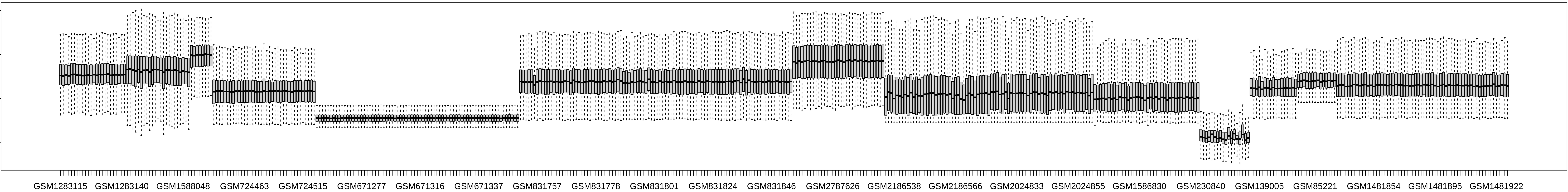

B

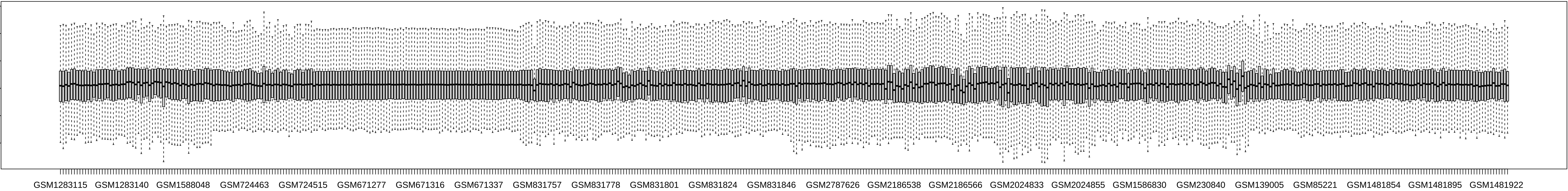

Supplementary Figure S1: Boxplots of 14 datasets. The range of log2 expression values for all samples is displayed before (A) and after (B) batch effect removal.
