# Supplementary material for: Amelioration for an ignored pitfall in reference gene selection by considering the mean expression and standard deviation of target genes
Source: Sci Rep. 2022 Jul 1;12:11129. doi: 10.1038/s41598-022-15277-5 (PMC9249883; doi:10.1038/s41598-022-15277-5)
Supplement: Supplementary file 2 — Supplementary Information 2. [file 41598_2022_15277_MOESM2_ESM.pdf]

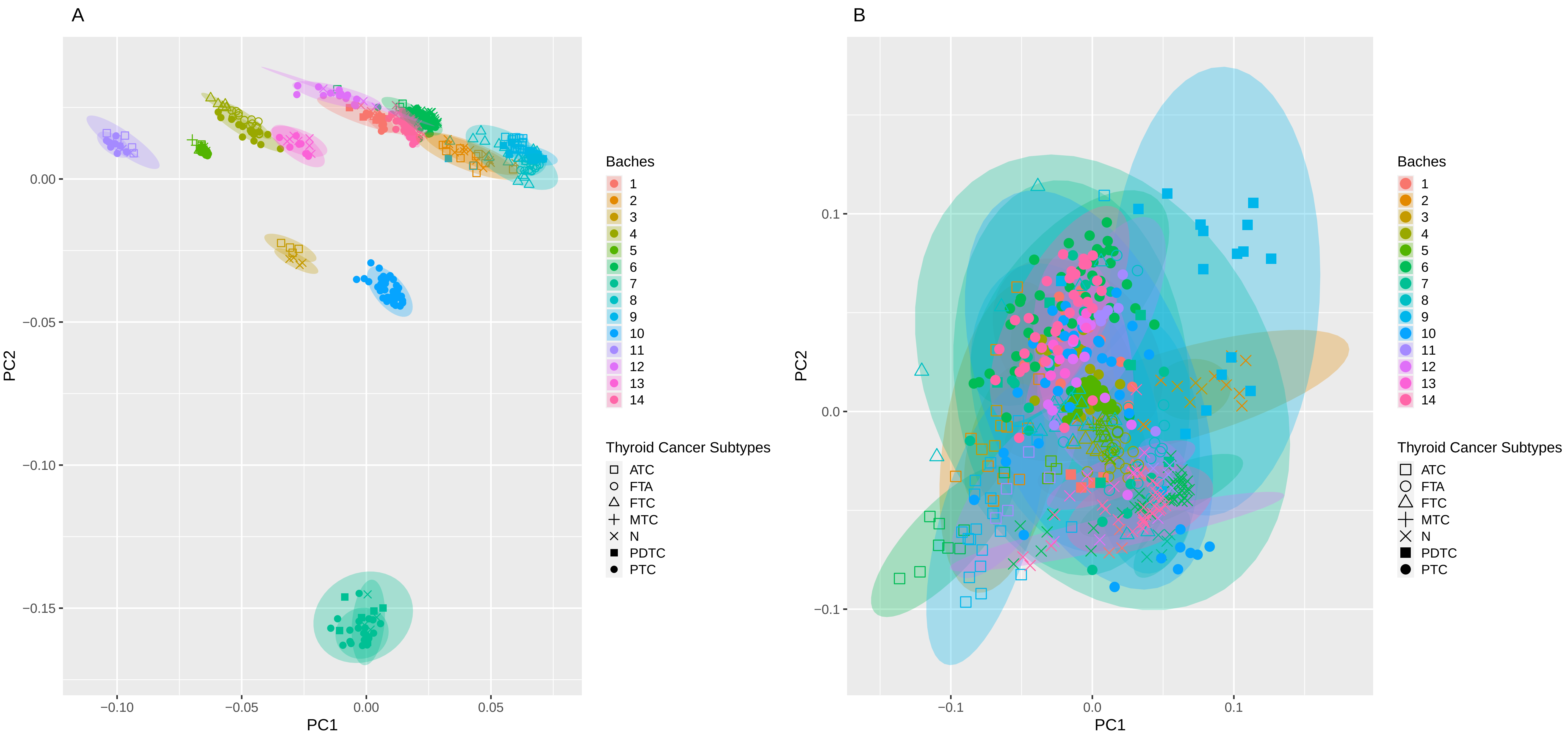

Figure S2: Principal component analysis (PCA) plots of the datasets before and after batch effect removal. Scatter plots show how samples are distributed depending on (A) batches or datasets before batch effect removal and (B) subtypes after batch effect removal.
