# Supplementary material for: Amelioration for an ignored pitfall in reference gene selection by considering the mean expression and standard deviation of target genes
Source: Sci Rep. 2022 Jul 1;12:11129. doi: 10.1038/s41598-022-15277-5 (PMC9249883; doi:10.1038/s41598-022-15277-5)
Supplement: Supplementary file 3 — Supplementary Information 3. [file 41598_2022_15277_MOESM3_ESM.pdf]

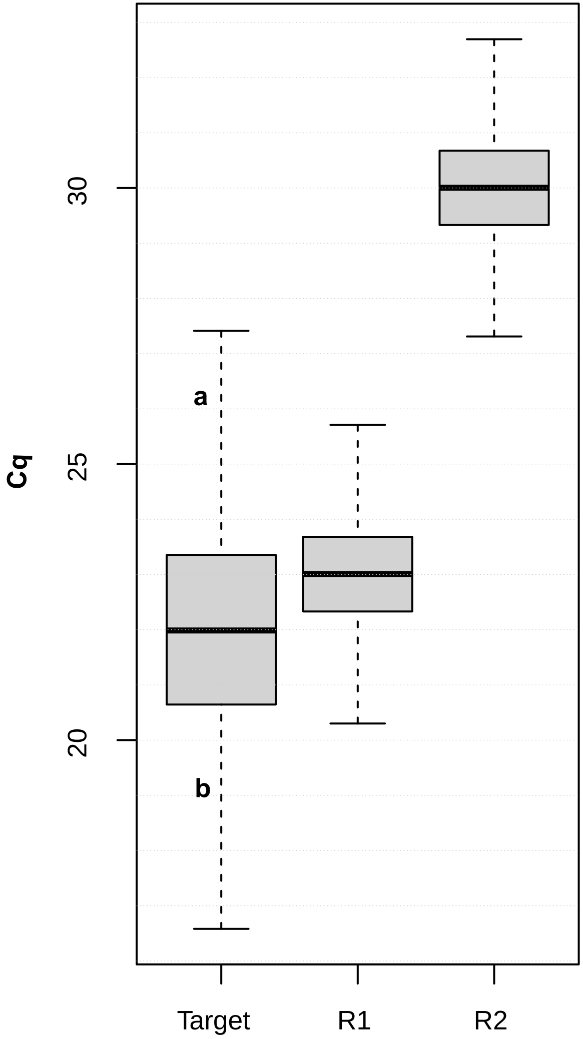

Figure S3: The expression of target gene (Target) is compared to that of reference genes 1 (R1) and 2 (R2). Target has an average Cq values of 22±2, R1 has an average Cq values of 23±1, and R2 has an average Cq values of 30±1. When normalized against R1, samples that express target gene in zone "a" have a positive delta-Cq (Cq target – Cq R1), whereas samples that express target gene in zone "b" have a negative delta-Cq. When the target gene is normalized to the R2 reference gene, which does not have overlapping Cq values with the target gene, the contradiction disappears.
