# Supplementary material for: Amelioration for an ignored pitfall in reference gene selection by considering the mean expression and standard deviation of target genes
Source: Sci Rep. 2022 Jul 1;12:11129. doi: 10.1038/s41598-022-15277-5 (PMC9249883; doi:10.1038/s41598-022-15277-5)
Supplement: Supplementary file 4 — Supplementary Information 4. [file 41598_2022_15277_MOESM4_ESM.pdf]

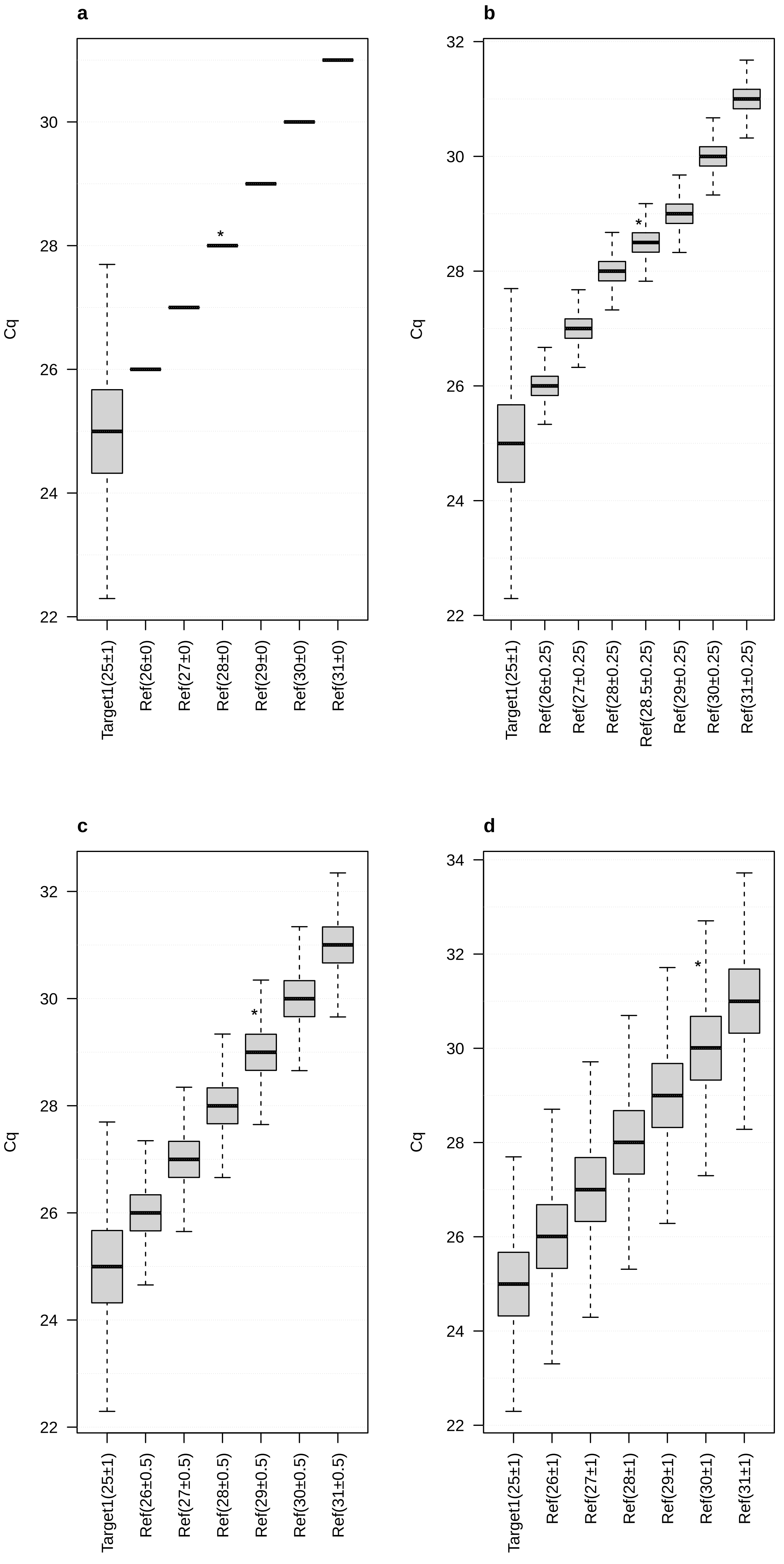

Figure S4: The target gene 1 was compared to reference genes with a variety of mean and SD values. (a) Reference genes with $\sigma=0$ do not show overlap with the target gene if $\mu_{reference} \geq 28$ (asterisk) and show overlap with $\mu_{reference} < 28$. (b) Reference genes with $\sigma=0.25$ do not show overlap with the target gene if $\mu_{reference} \geq 28.5$ (asterisk) and show overlap with $\mu_{reference} < 28$. (c) Reference genes with $\sigma=0.5$ do not show overlap with the target gene if $\mu_{reference} \geq 29$ (asterisk) and show overlap with $\mu_{reference} < 29$. (d) Reference genes with same SD as the target gene ($\sigma=1$) do not show overlap with the target gene if $\mu_{reference} \geq 30$ (asterisk) and show overlap with $\mu_{reference} < 30$.
