# Supplementary material for: Amelioration for an ignored pitfall in reference gene selection by considering the mean expression and standard deviation of target genes
Source: Sci Rep. 2022 Jul 1;12:11129. doi: 10.1038/s41598-022-15277-5 (PMC9249883; doi:10.1038/s41598-022-15277-5)
Supplement: Supplementary file 5 — Supplementary Information 5. [file 41598_2022_15277_MOESM5_ESM.pdf]

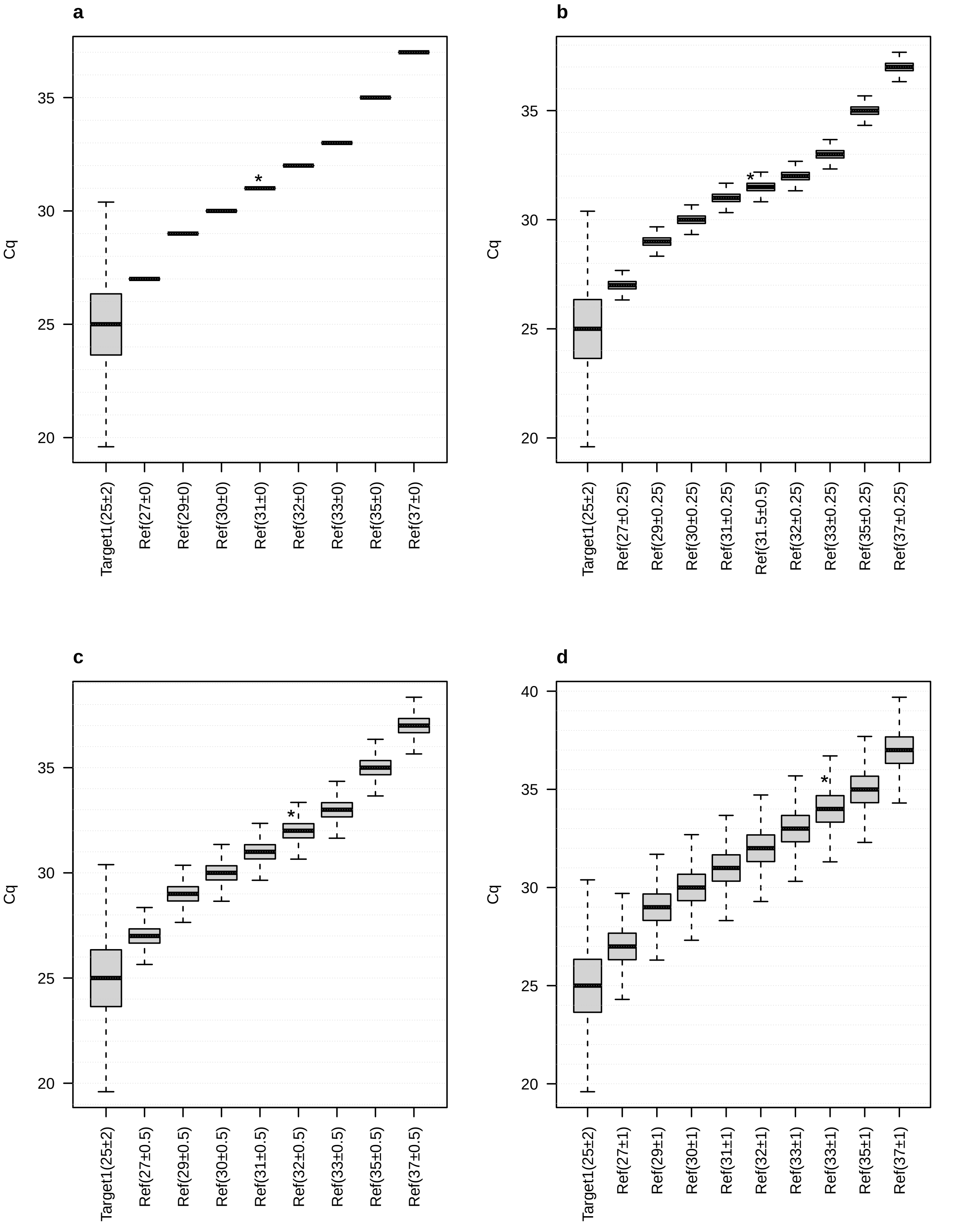

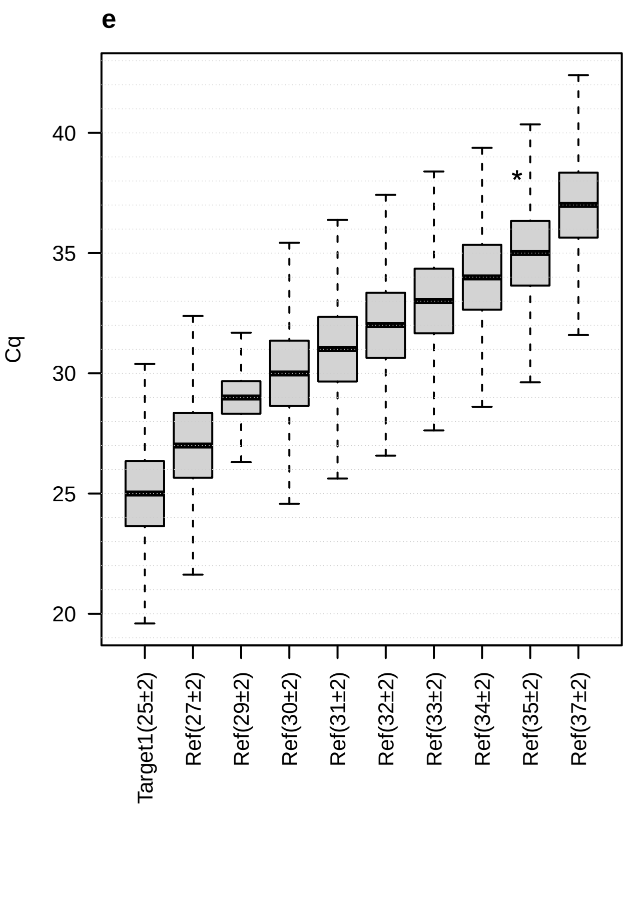

Figure S5: The target gene 2 was compared to reference genes with a variety of mean and SD values. (a) Reference genes with σ=0 do not show overlap with the target gene if $\mu_{reference} \geq 31$ (asterisk) and show overlap with $\mu_{reference} < 31$. (b) Reference genes with σ=0.25 do not show overlap with the target gene if $\mu_{reference} \geq 31.5$ (asterisk) and show overlap with $\mu_{reference} < 31.5$. (c) Reference genes with σ=0.5 do not show overlap with the target gene if $\mu_{reference} \geq 32$ (asterisk) and show overlap with $\mu_{reference} < 32$. (d) Reference genes with same SD as the target gene (σ=1) do not show overlap with the target gene if $\mu_{reference} \geq 33$ (asterisk) and show overlap with $\mu_{reference} < 33$. e) Reference genes with same SD as the target gene (σ=1) do not show overlap with the target gene if $\mu_{reference} \geq 35$ (asterisk) and show overlap with $\mu_{reference} < 35$.
