# Supplementary material for: Amelioration for an ignored pitfall in reference gene selection by considering the mean expression and standard deviation of target genes
Source: Sci Rep. 2022 Jul 1;12:11129. doi: 10.1038/s41598-022-15277-5 (PMC9249883; doi:10.1038/s41598-022-15277-5)

**Supplementary information for Amelioration for an ignored pitfall in reference gene selection by considering the mean expression and standard deviation of target genes.**

Ghazal Esfandiarpour^1^, Mohammad Mokhtari^2^, Seyed-Morteza Javadirad*^1^, Mohsen Kolahdouzan^3^, Ahmed Almuslimawi^1^

**Supplementary Table S1: Genes and primers used in this study**

| **Gene symbol**  **(Accession number)** | **Full name** | **Forward (F) and reverse (R) primers** | **Product length (bp)** | **Annealing temperature (°C)** |
| --- | --- | --- | --- | --- |
| *GAPDH* (NM_001256799.3) | Glyceraldehyde-3-phosphate dehydrogenase | F:CCACTCCTCCACCTTTGACG  R:CCACCACCCTGTTGCTGTAG | 107 | 58 |
| *SYMPK* (NM_004819.3) | Symplekin | F:ACGGTGCTGAGGGTCATTGA  R:GAGGGTGGGACTTTGTCTGTGA | 146 | 60 |
| *NKX2-1*  (NG_013365.1) | NK2 Homeobox 1 | F:CAGGACACCATGAGGAACAGCG  R:GCCATGTTCTTGCTCACGTCCCG | 151 | 60 |
| *RTRAF*  (NM_016039) | RNA Transcription, Translation And Transport Factor | F:TGGTTTTGACACAGGAGATGC  R:AACAGCTACTATGGCTTCGTTG | 115 | 60 |
| *ETS1*  (NM_005238.4) | ETS proto-oncogene 1, transcription factor | F:CGATCTCAAGCCGACTCTCAC  R:GGGACATCTGCACATTCCATATCCG | 90 | 58 |

Genes and their corresponding primers are listed in great detail. The GeneBank accession number, forward (F) and reverse (R) primer sequences, product length and primer annealing temperature are listed.

**Supplementary Table S2: Detailed information of microarray datasets.**

| **Dataset Number** | **Accession Number** | **Tissue Samples** | **Platform** | **Country** | **Outliers** |
| --- | --- | --- | --- | --- | --- |
| 1. | GSE53157 | 27 samples: (5 PDTC, 7 cPTC,8 fvPTC, 4 FTC,3 normal ) | GPL570 | Portugal | 3 samples |
| 2. | GSE65144 | 25 samples: (12 ATC, 13 normal) | GPL570 | USA | 2 samples |
| 3. | GSE53072 | 9 samples: (5 ATC, 4 normal ) | GPL6244 | Portugal | 1 samples |
| 4. | GSE29315 | 71 samples: (9 Hurthle cell adenomas, 17 FA, 9 FTC, 13 FVPTC,6 Hashimoto thyroiditis,8 thyroid hyperplasias,9 PTC) | GPL8300 | Belgium | 34 samples: (including all hurthle cell adenomas, Hashimoto thyroiditis and thyroid hyperplasias) |
| 5. | GSE27155 | 99 samples: (4 normal,10 FTA,13 FTC,7 oncocytic adenomas,8 oncocytic carcinomas,51 PTC,4 ATC, and 2 MTC) | GPL96 | USA | 26 samples: (including all oncocytic adenomas and oncocytic carcinomas) |
| 6. | GSE33630 | 105 samples: (11 ATC, 49 PTC,45 normal) | GPL570 | Belgium | 7 samples |
| 7. | GSE104006 | 34 samples: (29 PTC, 5 normal) | GPL14951 | Italy | 1 samples |
| 8. | GSE82208 | 52 samples: (27 FTC, 25 FTA) | GPL570 | Poland | 14 samples |
| 9. | GSE76039 | 37 samples: (20 ATC, 17 PDTC) | GPL570 | USA | - |
| 10. | GSE65074 | 38 samples: (PTC with different stages) | GPL13667 | Denmark | - |
| 11. | GSE9115 | 19 samples: (4 normal, 10 PTC, 5 ATC) | GPL5917 | USA | 1 sample |
| 12. | GSE6004 | 18 samples: (4 normal, 14 PTC) | GPL570 | USA | 1 sample |
| 13. | GSE3678 | 14 samples: (7 normal, 7 PTC) | GPL570 | USA | - |
| 14. | GSE60542 | 92 samples: (29 lymph node, 63 normal or PTC) | GPL570 | Belgium | 30 sample |

Microarray datasets are listed in detail, including accession numbers, tissue samples, platform, nation and probable outliers.

PTC: papillary thyroid carcinoma, PDTC: poorly differentiated thyroid cancer, cPTC: classical papillary thyroid carcinoma, FVPTC: follicular variant of papillary thyroid carcinoma, FTC: follicular thyroid carcinoma, ATC: anaplastic thyroid carcinoma, FTA: follicular thyroid adenoma, MTC: medullary thyroid carcinoma

Supplementary Figure S1


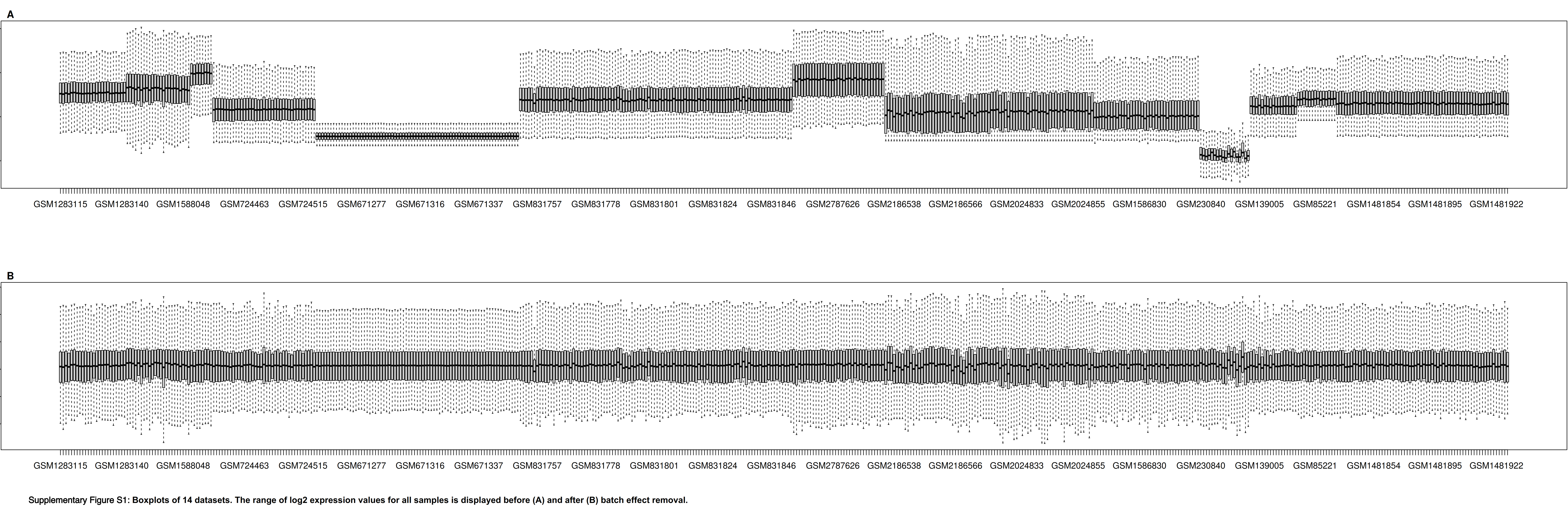


Supplementary Figure S2


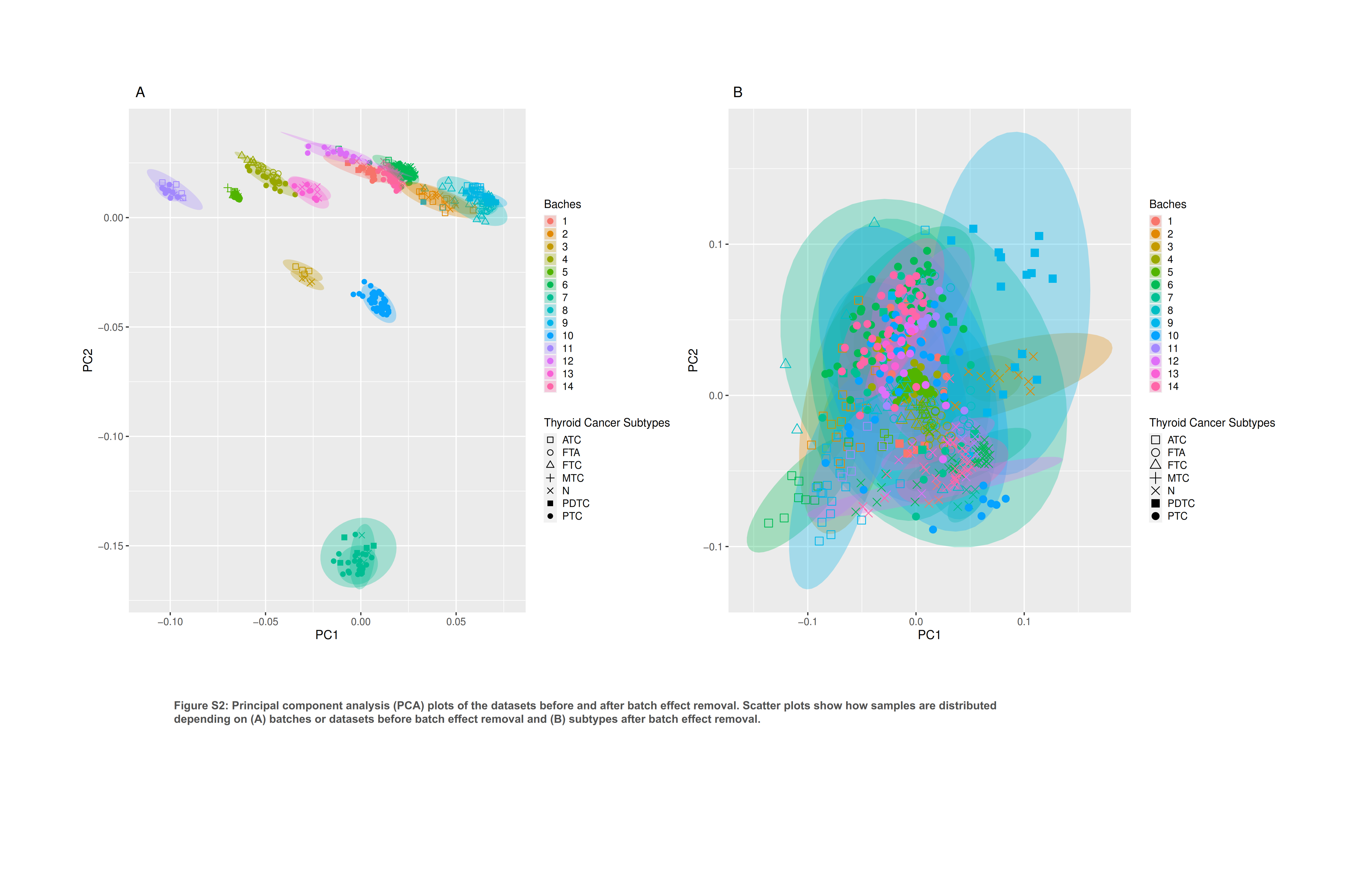

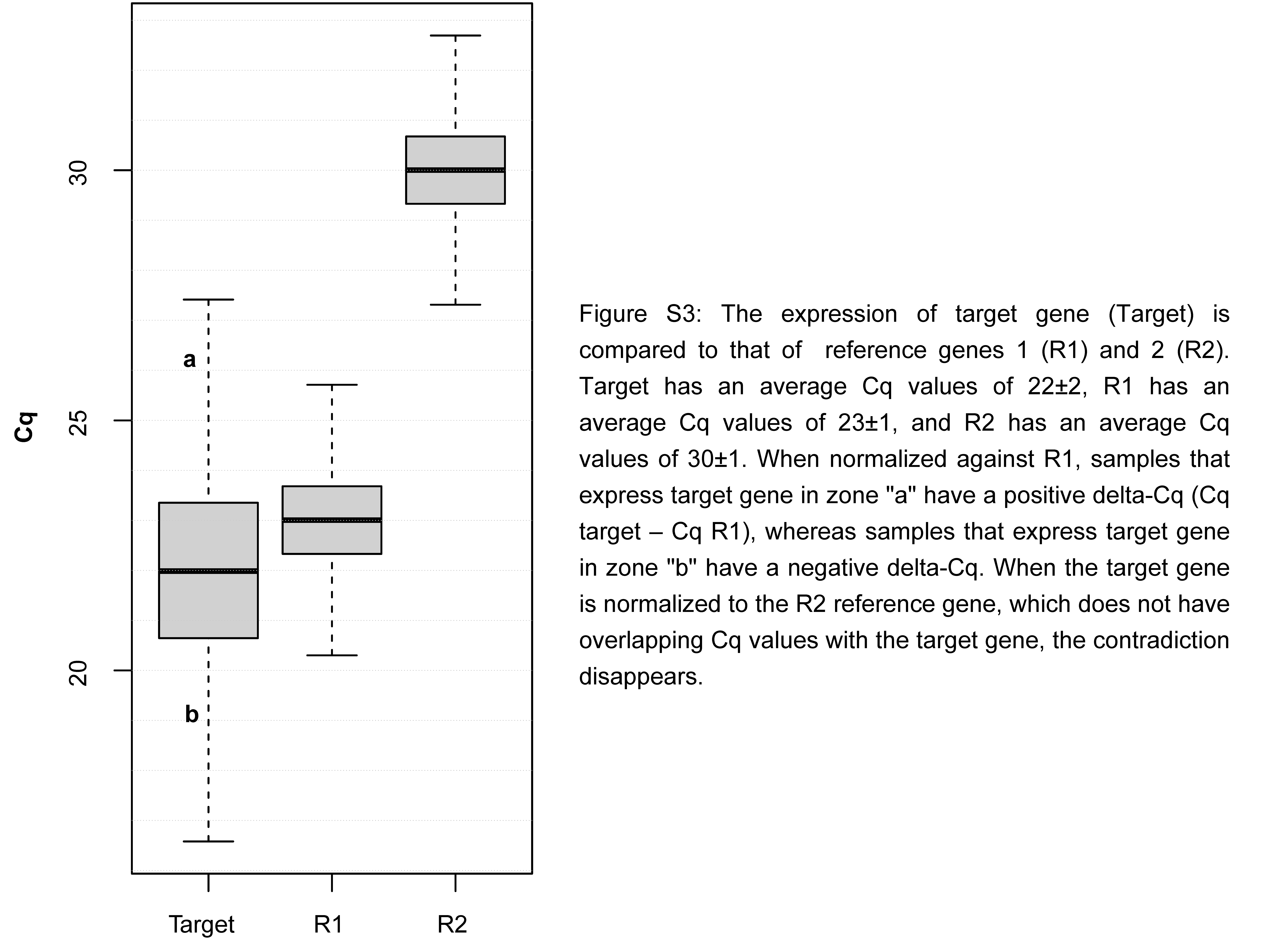

Supplement: Supplementary file 6 — Supplementary Information 6. [file 41598_2022_15277_MOESM6_ESM.docx]
